# From Clipping to Coiling and Beyond: Mapping the Evolution and Future Directions of Intracranial Aneurysm Research

**DOI:** 10.3390/brainsci16090984

**Published:** 2026-09-16

**Authors:** Jure Pešak, Andrej Kastrin

**Affiliations:** 1 Department of Neurosurgery, University Medical Centre Ljubljana, 1000 Ljubljana, Slovenia; jure.pesak@kclj.si; 2 Interdisciplinary Doctoral Programme in Biomedicine, University of Ljubljana, 1000 Ljubljana, Slovenia; 3 Institute for Biostatistics and Medical Informatics, Faculty of Medicine, University of Ljubljana, 1000 Ljubljana, Slovenia

**Keywords:** intracranial aneurysm management, endovascular treatment, microsurgical clipping, aneurysm hemodynamics and biomechanics, bibliometrics

## Abstract

**Highlights:**

**What are the main findings?**

- Co-word analysis of 22,061 PubMed publications (1946–2023) identified four distinct phases of intracranial aneurysm research: open-surgery, transitional, endovascular, and multidisciplinary, each characterized by shifts in dominant MeSH-term clusters.

**What are the implications of the main findings?**

- The field’s thematic trajectory reflects a fundamental transition from reactive, technique-driven management of ruptured aneurysms to predictive, individualized risk stratification for unruptured lesions.
- The mapped conceptual structure highlights persistent research gaps, including limited representation of mechanistic and basic-science research and continued reliance on observational rather than randomized evidence.

**Abstract:**

**Background/Objectives**: Intracranial aneurysm (IA) management has evolved from surgical treatment of ruptured lesions into a multidisciplinary field integrating diagnostics, neurocritical care, endovascular techniques, and individualized risk assessment. As the literature has grown beyond the scope of traditional narrative synthesis, bibliometric methods are needed to characterize the field’s development. We used co-word analysis to map the conceptual structure and thematic evolution of IA research over nearly eight decades. **Methods**: We analyzed 22,061 publications indexed in PubMed between 1946 and 2023. Co-word analysis of Medical Subject Headings tracked thematic development across six subperiods grouped into eras. Within each thematic network, topics were classified by centrality and density as motor, niche (highly developed but isolated), emerging or declining, or basic themes. **Results**: Four historical eras were delineated as an interpretative synthesis of the six subperiods. The open-surgery era (1946–1980) centered on ruptured aneurysms and refinement of operative techniques. The transitional era (1981–1990) was marked by proactive vasospasm management and emerging neurocritical care. The endovascular era (1991–2010) reflected the rise of evidence-based medicine and endovascular therapy. The multidisciplinary era (2011–2023) shifted toward unruptured aneurysms, molecular research, biomechanics, and individualized risk stratification. Recent themes, including hemodynamics, flow diversion, and precision medicine, reflect a growing research emphasis on predictive and personalized approaches to aneurysm management. **Conclusions**: IA research has evolved from descriptive, technique-oriented studies toward a data-driven landscape increasingly focused on predictive assessment and management of unruptured lesions. Its thematic evolution highlights the growing prominence of molecular and computational approaches, although their clinical and translational impact cannot be directly inferred from the present analysis.

## 1. Introduction

Unruptured intracranial aneurysms (IAs) are localized dilations of cerebral arteries, with an estimated prevalence of approximately 2% among adults without risk factors for subarachnoid hemorrhage (SAH), a figure that increases with age [1]. Structural weakening of the aneurysmal wall, driven by histomorphological remodeling, predisposes the vessel to rupture under hemodynamic stress [2,3]. Currently, risk stratification is guided by the Population, Hypertension, Age, Size of aneurysm, Earlier SAH, Site of aneurysm (PHASES) score [4]. While the mean five-year rupture risk is estimated at 3.4%, the risk associated with prophylactic interventions, whether endovascular or microsurgical, can reach 5%. Consequently, careful individualization is paramount for effective management. Once rupture occurs, aneurysmal SAH carries high morbidity and mortality; only half of the affected patients regain functional independence following treatment [5]. The strongest predictor of clinical outcome remains the neurological condition at presentation, which underscores the necessity for timely management of this pathology [6].

Historically, aneurysm treatment has evolved alongside advances in neurosurgery and vascular physiology, necessitated by the high risk of rupture and a narrow therapeutic window [7,8]. While awareness of arterial aneurysms dates back to antiquity, with references found in Egyptian surgical papyri, systematic interventions emerged much later. In 1785, Hunter laid the foundation for targeted surgical approaches by introducing proximal ligation for peripheral aneurysms. Horsley adapted this technique for intracranial use in 1885, performing carotid artery ligation to manage cerebral aneurysms. In the early 20th century, Cushing innovated various techniques, such as silver clip placement and wrapping, which substantially advanced vascular neurosurgery. A major milestone occurred in 1937 when Dandy [9] performed the first direct surgical clipping of a posterior communicating artery aneurysm via a pterional approach (i.e., frontotemporal craniotomy). This breakthrough, coupled with Yasargil’s [10,11] refinement of microneurosurgical techniques in the late 1960s, established the foundation of modern aneurysm surgery.

Given the broad thematic scope of contemporary IA research, there is a burgeoning need for a systematic synthesis of existing knowledge [12]. This task is complicated by the exponential expansion of scientific literature. Bibliometric analysis, the quantitative study of publications using metrics such as volume, citation patterns, and collaboration networks, provides a systematic framework to examine research activity and impact [13]. Beyond descriptive measures, bibliometric approaches facilitate science mapping, specifically through co-word analysis. By examining the co-occurrence of keywords, this method identifies conceptual relationships and traces thematic development over time [14]. These approaches enable the identification of dominant themes, knowledge gaps, and emerging frontiers, revealing the underlying architecture of scientific knowledge.

Previous bibliometric evaluations of IA literature have primarily focused on performance indicators, such as citation impact and author rankings. While these metrics offer an overview of productivity, they do not capture the intellectual structure or the thematic transitions within the domain. Without a systematic examination of conceptual associations, the evolution of IA research remains largely unmapped. This limitation impedes the integration of scientific insights, particularly regarding diagnostic innovation and therapeutic shifts. Given the increasingly intricate nature of neurosurgical literature, a specialized science-mapping analysis is essential to clarify the field’s thematic pathways and identify emerging frontiers.

To address these gaps, the present study employs co-word analysis to delineate the thematic structure and evolution of IA research. Utilizing a comprehensive dataset from the MEDLINE/PubMed database, we aim to: (i) map the conceptual structure of the field to identify core research themes; (ii) identify temporal trends, capturing both historical trajectories and recent technological developments; and (iii) provide an expert-driven interpretation of thematic clusters to contextualize structural dynamics and highlight persistent knowledge gaps in IA research.

## 2. Related Work

Existing bibliometric literature on IAs has primarily focused on classical indicators, including global research productivity, author collaboration, and citation patterns. Taken together, these studies provide essential perspectives on the scientific footprint, citation impact, and global distribution of research activity. Kiraz et al. [12] provided a foundational macro-scale overview by analyzing more than 13,000 IA-related articles published between 1980 and 2019. Their work characterizes the dominant contributing authors, journals, institutions, and countries, demonstrating a steady increase in publication volume and a period of sustained expansion since the 1980s. Parallel to the growth of the evidence base, the organizational scale of research has undergone a significant transition. Assessments of high-impact literature have identified a notable shift from single-center reports toward large-scale, multi-institutional collaborations [15]. Furthermore, longitudinal evaluations of the last decade identify a rise in international research partnerships and the increasing connectivity of global co-authorship networks [16].

Bibliometric studies reflect a transition in clinical paradigms, shifting from conventional microsurgical techniques to endovascular interventions. Lu et al. [15] empirically revealed this trend by analyzing the 100 most-cited articles in the field, demonstrating the current dominance of endovascular techniques over traditional surgical standards. This evolution is further supported by Zhang et al. [16], who identify endovascular coiling and flow diversion as the primary research hotspots of the last decade. Notably, the rise of minimally invasive techniques has not rendered open surgery obsolete; Qin et al. [17] underscore the ongoing importance of microsurgical clipping, particularly for aneurysms with complex morphologies that remain challenging for endovascular treatment. However, technological advancements have not always been matched by corresponding clinical validation. Fu et al. [18] observe that the proliferation of reports on innovative endovascular procedures far exceeds the accumulation of high-level empirical evidence, indicating an imbalance between technological progress and clinical evaluation.

Expanding from macroscopic clinical trends, the bibliometric literature also characterizes discrete clinical domains and focused research niches. Chen et al. [19] traced the global evolution of animal model research, highlighting a shift from traditional surgical validation toward molecular investigations into macrophage-mediated inflammation and elastase-induced models of arterial wall remodeling. Furthermore, targeted analyses have synthesized evidence within therapeutically specific areas, such as the identification of landmark publications in basilar artery aneurysm management [20] and the emerging research landscape of intracranial nicardipine for post-aneurysmal vasospasm [21]. These studies provide granular insights into both the mechanistic underpinnings of IA pathology and the unique management challenges posed by high-risk anatomical subsets.

Complementing the developments in interventional techniques, the diagnostic and analytical framework of IA research has also expanded, shifting from retrospective clinical reporting toward advanced predictive modeling and imaging-based risk stratification. Chen et al. [22] utilize bibliometric mapping to demonstrate that hemodynamics research has evolved from foundational flow analyses toward frontier topics such as four-dimensional flow magnetic resonance imaging (4D-flow MRI) and morphology-based rupture prediction. This movement toward precision medicine is increasingly driven by data-centric methodologies; for instance, Zhang et al. [23] identify artificial intelligence (AI) as a primary research hotspot, with an emphasis on computer-assisted diagnosis and individualized risk assessment. Their work highlights a broader transition in clinical decision-making, moving from population-level guidelines toward personalized frameworks that integrate multi-dimensional data for improved prognostic accuracy.

In summary, the existing bibliometric landscape reveals a field undergoing a multifaceted transition from open surgery to endovascular therapy, and from descriptive clinical observations toward molecular-level investigation and AI-driven predictive modeling. While previous studies have provided snapshots of individual niches, the rapid pace of innovation necessitates a systematic synthesis that integrates these diverse threads into a single longitudinal framework. The present study seeks to bridge the gap between historical paradigms and the current frontier by providing a unified analysis of the global IA research community.

## 3. Materials and Methods

This study uses co-word analysis to examine the thematic structure of scientific literature on IA research published from 1946 to 2023. The Methods section follows the analytical workflow and is divided into two main phases. First, we describe the literature search strategy and the retrieval of bibliographic records. Next, we detail the science mapping procedure, including the co-word analysis and the use of thematic mapping indicators as the interpretive framework for characterizing the research landscape and clinical priorities.

Ethical approval and patient consent were not required for this study because it is a bibliometric analysis based on aggregated data retrieved from publicly available databases.

All analyses were performed in R (v4.6.1) using the bibliometrix package (v5.4.1) [24]. In overview, the analysis pipeline (i) retrieves and parses PubMed records; (ii) divides the study period into consecutive time periods and, for each period, constructs a Medical Subject Headings (MeSH) term co-occurrence network normalized using the association-strength (equivalence) index; (iii) partitions each network into thematic clusters using the Louvain algorithm; and (iv) characterizes the resulting clusters using Callon’s centrality and density measures. The alluvial diagram tracing thematic evolution across periods was constructed from period-to-period cluster inclusion indices and visualized using TikZ [25]. The complete programming code used to retrieve the data and reproduce all figures and tables is publicly available in the GitHub repository at https://github.com/akastrin/neurobib (commit sha 6e19d0f, accessed on 11 September 2026).

### 3.1. Data Collection

The literature search and retrieval were conducted using MEDLINE/PubMed, a premier bibliographic database of the US National Library of Medicine (NLM). A notable feature of MEDLINE is that its records from 1946 onward are routinely indexed using NLM MeSH. Unlike author keywords, MeSH terms are standardized and assigned by trained indexers to annotate MEDLINE records, ensuring consistency and high-quality content representation.

We targeted publications in which IA was the primary research focus. The PubMed search was limited to peer-reviewed journal articles published in English and indexed in the MEDLINE subset. Publications were identified using the search query (intracranial aneurysm [MAJR] OR intracranial aneurysm [TI]) AND medline [SB] AND english [LA] AND journal article [PT]. The search query was formulated based on a preliminary review of the existing literature and consultation with domain experts.

To maintain an objective and reproducible dataset, manual title and abstract screening was omitted in favor of a high-precision search strategy. This protocol inherently limited the dataset to highly relevant literature. Because major MeSH terms denote the primary focus of a study and article titles typically reflect the central subject matter, this targeted configuration ensured high conceptual relevance while minimizing subjective researcher bias.

### 3.2. Data Analysis

**Figure 1:**
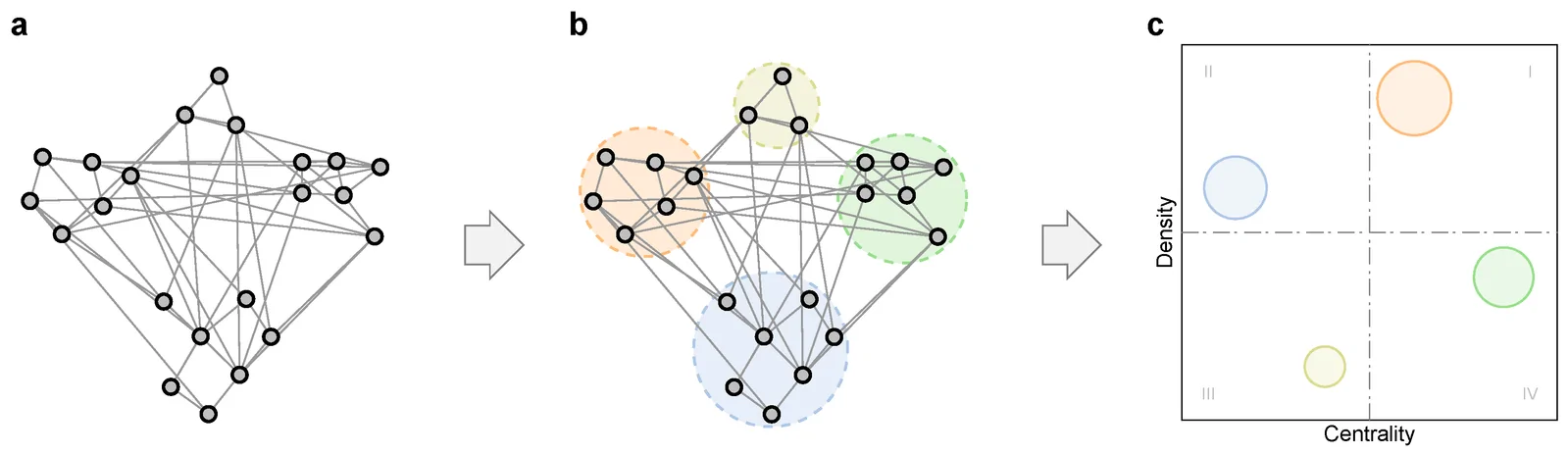
Computational workflow for transforming MeSH term co-occurrence data into thematic clusters and their strategic mapping. (**a**) Construction of the MeSH term co-occurrence network from the retrieved publication set. Nodes correspond to individual terms, and edges indicate their co-occurrence within the same publication. (**b**) Partitioning of the co-occurrence network into term clusters using the Louvain community detection algorithm. Edges connecting terms assigned to the same cluster are referred to as internal links, whereas edges connecting terms from different clusters are referred to as external links; internal links contribute to cluster density and external links to cluster centrality. Circle colors are assigned arbitrarily and indicate cluster membership only. (**c**) Projection of the identified clusters onto a strategic diagram according to Callon’s centrality and density measures, reflecting inter-cluster connectedness (external links) and internal cohesion (internal links), respectively. Bubble size is proportional to the number of unique terms within each cluster. See text for further details.

To gain insight into the conceptual structure of the field, we used co-word analysis to extract individual research topics and trace their thematic evolution (Figure 1). In this approach, each publication is represented by its assigned set of MeSH descriptors. These terms form the nodes of a thematic network, where edges represent the co-occurrence of MeSH terms within the same publication (Step 1). In the resulting network, node size reflects the frequency of the corresponding MeSH term in the corpus, while edge weight represents the strength of their conceptual association. To account for differences in term frequencies and highlight significant links, the raw co-occurrence counts were normalized using the association strength (equivalence index), defined as $e_{ij}=c_{ij}^{2}/\left(c_{i}c_{j}\right)$, where $c_{ij}$ is the number of bibliographic records in which two MeSH terms, *i* and *j*, co-occur, and $c_{i}$ and $c_{j}$ are the total occurrences of each term. The normalized co-occurrence count ranges from zero, if the MeSH pair never co-occurs, to one, if they appear together in every record.

In Step 2, we applied a clustering algorithm to organize MeSH terms into thematic subfields, capturing close conceptual relationships between terms and delineating the structure of the research field. Specifically, we used the Louvain community detection algorithm [26]. The Louvain algorithm is a greedy, multi-level modularity-optimization method: it first assigns each node to its own community and iteratively moves nodes to neighboring communities when doing so increases network modularity; once no further local improvement is possible, the resulting communities are aggregated into “super-nodes”, and the process is repeated on the resulting network until modularity can no longer be improved. We selected Louvain clustering because it scales efficiently to the size of our co-occurrence networks and requires no prior specification of the number of clusters.

Finally, in Step 3, we characterized the extracted clusters using a co-word approach based on the work of Callon et al. [14]. Co-word analysis utilizes two measures, centrality and density, to map a specific cluster into a two-dimensional vector space. Centrality is defined as $c=10\sum e_{kh}$, where *k* is a MeSH term from the target cluster, *h* is a MeSH term from another cluster, and $e_{kh}$ is the normalized co-occurrence frequency of both terms, *k* and *h*, as defined above. Centrality represents the relatedness of a given cluster to other clusters; in other words, it expresses the degree of importance or “prestige” of a research topic within the entire scientific field.

Density is given by $d=100(\sum e_{ij})/w$, where *i* and *j* are MeSH terms associated with the same cluster, and $e_{ij}$ is the normalized number of co-occurrences between them. Here, *w* refers to the total number of terms within the cluster. Density represents the internal cohesion of the cluster, reflecting the level of conceptual development and maturity of the research topic. The multiplicative constants 10 and 100 follow the original scaling convention introduced by Callon et al. [14] and widely retained in subsequent co-word analyses; they rescale the underlying association-strength sums, which are otherwise small fractional values, onto a more readily interpretable numeric range and do not carry additional substantive meaning. Because both centrality and density are subsequently normalized to the 0–1 range for the strategic diagrams, the specific choice of scaling constant does not influence cluster positions or their classification into quadrants.

Considering both of Callon’s measures, we can create a strategic diagram to represent the structural landscape of a given research area. In a strategic diagram, the *x*-axis represents centrality and the *y*-axis represents density. The plot is centered at the median values of both axes, dividing the diagram into four quadrants representing different types of research behavior. Each cluster can be qualitatively interpreted based on the quadrant in which it appears, as follows:

1. Motor topics: Clusters in Quadrant I are characterized by high centrality and high density. These clusters are well-developed and have typically been studied over long periods by established research groups.
2. Niche topics: Clusters in Quadrant II have low centrality but high density. These clusters are highly homogeneous, with strong internal connections, but remain isolated from the rest of the field due to weak external links.
3. Emerging or declining topics: Clusters in Quadrant III have both low centrality and low density, representing either nascent (emerging) or fading research topics.
4. Basic topics: Clusters in Quadrant IV have high centrality and low density, encompassing transversal and general research topics. While important for the research community, these topics are not yet internally well-developed.

Quadrant position alone cannot distinguish an emerging from a declining topic, as both occupy the same low-centrality, low-density region of the strategic diagram (“peripheral” quadrant). Thus, quadrant position identifies peripheral themes, while their temporal trajectories across successive periods distinguish emerging from declining themes.

The size of each cluster in the strategic diagram is proportional to the number of publications assigned to that thematic area. All terms within a cluster are sorted by frequency, and the most frequent term is selected as the representative label for that cluster.

## 4. Results

**Figure 2:**
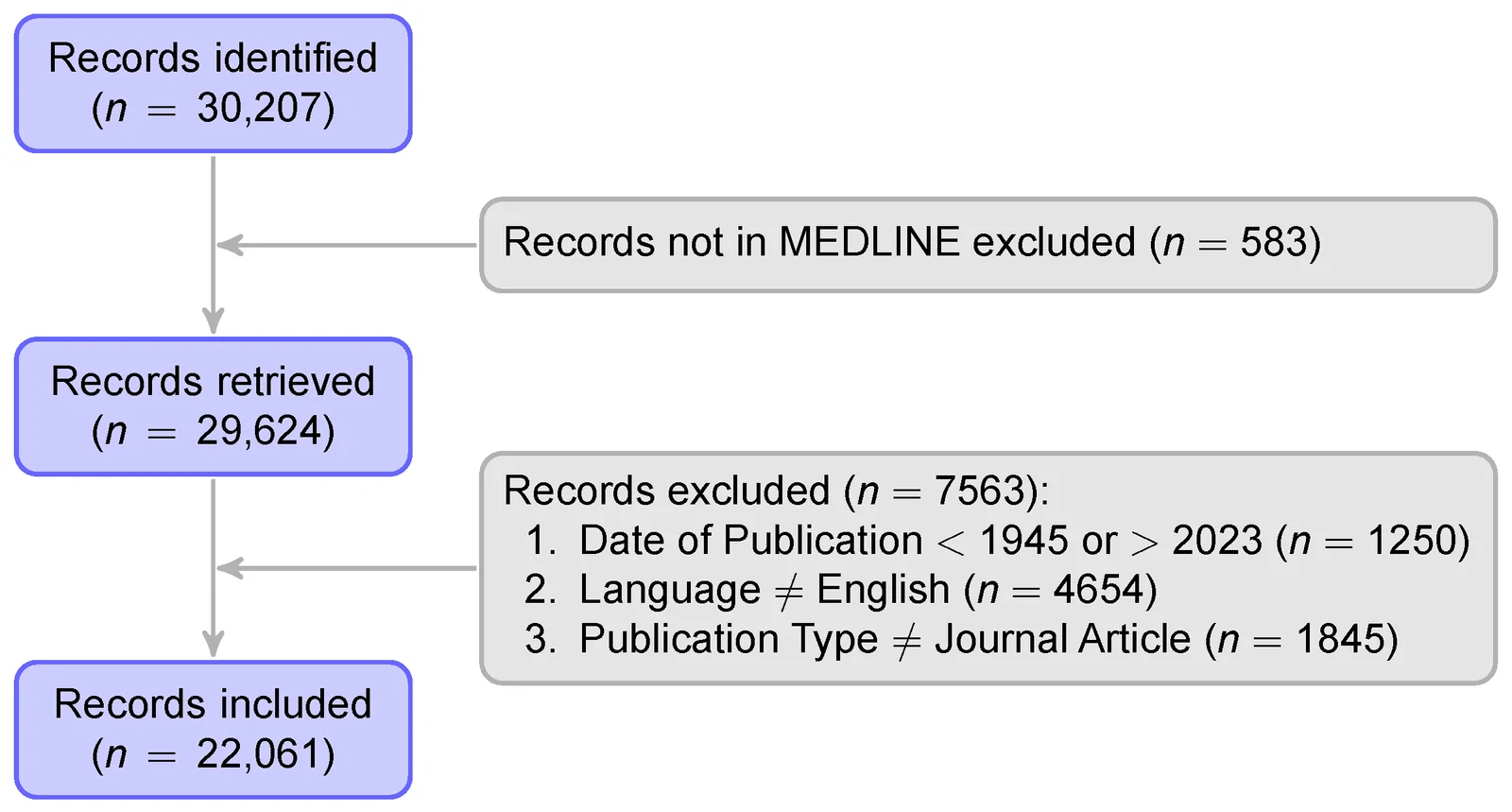
Flowchart of the study selection process.

A systematic literature search of PubMed was conducted from its inception through 31 December 2023. The search was last updated on 15 July 2025. The paper selection process is shown in Figure 2. Final co-word analysis was performed on 22,061 bibliographic records published between 1946 and 2023. We first describe the basic bibliometric characteristics of the collected corpus and then examine the thematic evolution of IA research across the study period.

### 4.1. Descriptive Bibliometric Analysis

The dataset shows a consistent upward trend in publication volume, with an annual growth rate of 7.67%. The mean document age is 17 years, indicating a field with a deep historical foundation and significant contemporary growth.

In terms of academic influence, the literature has received an average of 26.89 citations per document (1715 citations per year), reflecting substantial and sustained scholarly engagement. The intellectual output results from contributions by 40,975 authors. While 726 researchers produced single-authored documents, the vast majority of the field’s knowledge base comes from collaborative efforts, as shown by an average of 5.81 co-authors per publication. Additionally, the global scope of IA research is evident in the 12.43% of documents resulting from international co-authorships, highlighting a well-connected international research network.

**Figure 3:**
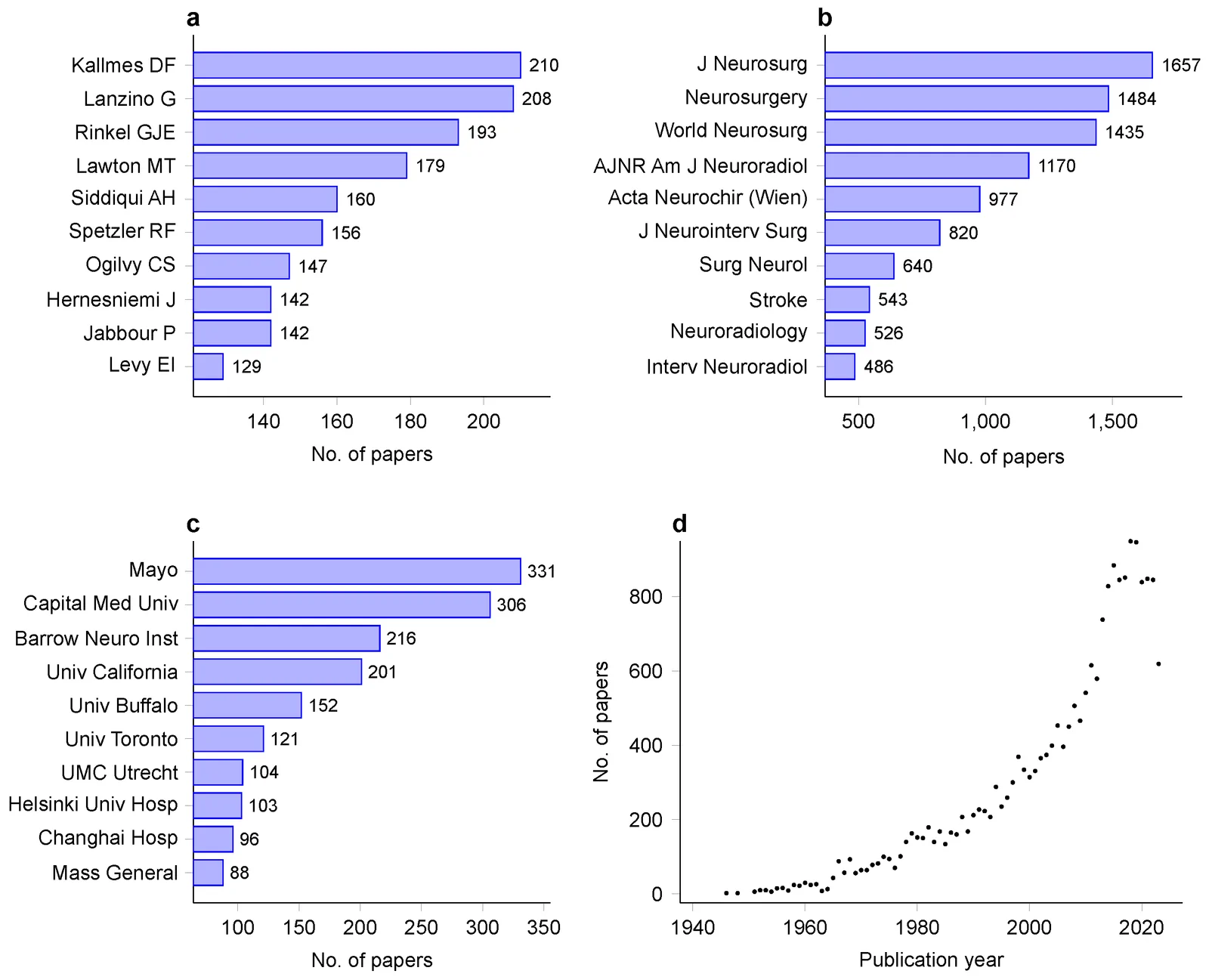
Global productivity metrics in IA research from 1946 to 2023 including (**a**) the top 10 researchers based on the number of authored papers, (**b**) the top 10 most frequent publication sources, (**c**) the top 10 most productive institutions based on the affiliation of the first author, and (**d**) the historical trajectory of annual publication volume.

Figure 3 summarizes global productivity metrics from 1946 to 2023. Panel (a) ranks the most prolific authors, showing that D.F. Kallmes is the leading contributor in the corpus with 210 publications, particularly in interventional neuroradiology, followed closely by G. Lanzino, a cerebrovascular neurosurgeon, and G.J.E. Rinkel, a neurologist specializing in SAH. Based on fractional authorship, which allocates credit by dividing a publication unit among co-authors to account for varying levels of contribution, R.F. Spetzler, a neurosurgeon specializing in cerebrovascular disease, ranks first. Panel (b) presents the distribution of publication venues, with the Journal of Neurosurgery leading in publication volume, followed by Neurosurgery and World Neurosurgery. Turning to institutional output, panel (c) identifies the Mayo Clinic (USA), Capital Medical University (China), and the Barrow Neurological Institute (USA) as the three most productive organizations. Finally, the historical trajectory shown in panel (d) indicates a sustained upward trend in the field, with a marked acceleration in research output since the mid-1980s.

To provide intellectual context for the thematic evolution of the field, a brief review of the most-cited papers in the corpus is presented in Table 1. These works provide the principal conceptual and clinical reference points for interpreting the science mapping results reported below. The ten most-cited articles predominantly addressed two major domains: unruptured IAs (four articles) and aneurysmal SAH or ruptured IAs (four articles), with the remaining two papers covering both ruptured and unruptured IAs (one article) and stroke more broadly (one article).

The most cited article is by the International Study of Unruptured Intracranial Aneurysms (ISUIA) investigators [27]. This prospective cohort study evaluated the natural history and treatment outcomes of unruptured IAs. An earlier study by the same research group, published in 1998, included both retrospective and prospective arms and laid the foundation for this prominent study [31]. In the same year, Rinkel et al. [1] presented an extensive systematic review, examining the prevalence of aneurysms in various retrospective and prospective studies and identifying novel risk factors for aneurysmal rupture. In 2011, Vlak et al. [33] published a systematic review estimating the prevalence of unruptured IAs based on different epidemiological parameters. In 2013, Becske et al. [35] reported on the pipeline embolization device as a novel technique in the endovascular field.

The second-most cited study, conducted by the International Subarachnoid Aneurysm Trial (ISAT) investigators in 2002, was a controversial clinical trial that compared endovascular and microsurgical treatments for ruptured IAs. This study had a major influence on contemporary clinical practice in the management of ruptured IA. The third-most cited study, authored by Hunt et al. [6] in 1968, was an observational study on the clinical classification of patients with aneurysmal SAH. Their work has been widely influential in neurosurgery, providing evidence on surgical risks related to the timing of intervention for ruptured IA. In 1990, Kassell et al. [30] published the first part of a study on the timing of ruptured IA surgery, emphasizing the importance of early and accurate diagnosis and management. In 2003, Raymond et al. [34] reported results on long-term angiographic recurrences after coiling both unruptured and ruptured IAs. Barber et al. [29] validated the Alberta Stroke Programme Early CT Score (ASPECTS) scale for thrombolytic therapy. This article is the only one among the top-cited list that is not directly related to IAs, but instead pertains to the field of stroke in general.

### 4.2. Science Mapping Analysis

To trace the evolution of the field over time, the dataset was divided into six time slices according to publication year (Table 2). The thematic maps for each period were generated using MeSH terms. To reduce noise and improve interpretability, we restricted the analysis to the top 200 most frequent MeSH terms in each period, requiring a minimum threshold of 3 occurrences per term. Consequently, 2871 documents were filtered out from the initial corpus of 22,061 records. The first time slice comprises 581 documents published up to and including 1970. The next four slices each span a decade: 1971–1980 (999 documents), 1981–1990 (1592 documents), 1991–2000 (2517 documents), and 2001–2010 (3889 documents). The final time slice covers 2011–2023 and includes 9612 documents. This distribution shows a substantial increase in publication volume over time, with approximately 97% of documents published after 1970. For each period, we provide a strategic diagram and its qualitative interpretation, including expert-driven historical and clinical contextualization based on the domain literature. Note that the centrality and density axes are normalized to the 0–1 range.

#### 4.2.1. Period 1946–1970

**Figure 4:**
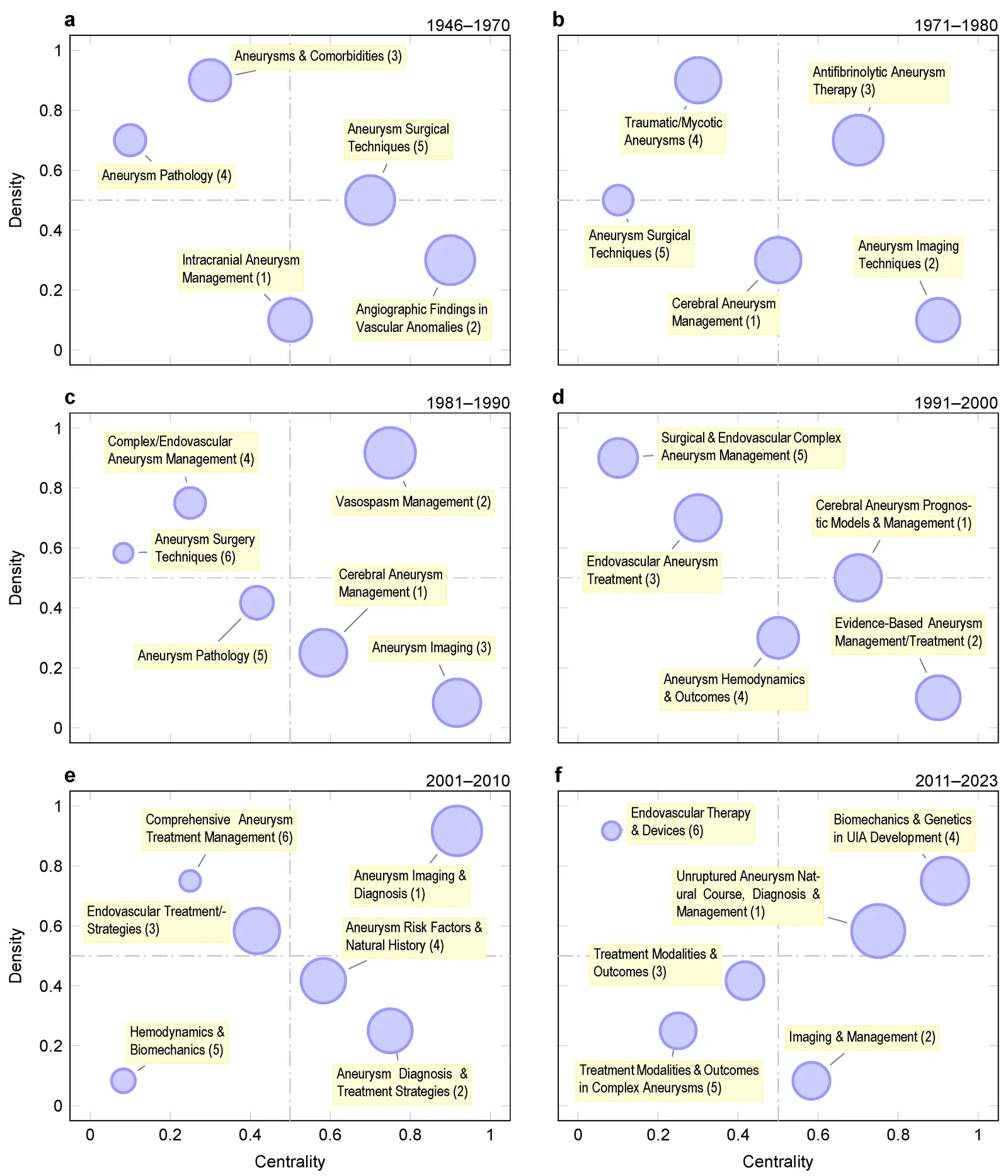
Temporal evolution of the research landscape based on co-word strategic diagrams. Panels (**a**–**f**) display the chronological shifts in the domain’s thematic structure across six time periods. Clusters are mapped by centrality (*x*-axis) and density (*y*-axis) to identify motor, basic, specialized, and emerging themes over time. Node sizes are proportional to the log-transformed total occurrence frequency of the MeSH terms within each cluster.

Figure 4a shows the time slice from 1946 to 1970. The cluster labeled “Angiographic Findings in Vascular Anomalies” reflects the basic research themes of this period. The clusters “Aneurysms & Comorbidities” and “Aneurysm Pathology” correspond to niche research areas; the former is more developed and well established, showing a trend toward the “hot topics” quadrant. The cluster “Intracranial Aneurysm Management” appears to be shifting from peripheral to basic relevance during this period, while “Angiographic Findings in Vascular Anomalies” is approaching a transition from a basic theme to a hot topic.

This period marked the beginning of IA research and was a pivotal moment in the field’s development. The focus was primarily on ruptured aneurysms, as they often presented with SAH. The cluster “Angiographic Findings in Vascular Anomalies” reflects the early adoption of cerebral angiography, which was the only reliable diagnostic method at the time. Tindall et al. [36] made significant contributions to understanding vascular hemodynamics in IAs and established angiography as the foundation of cerebrovascular diagnosis. Surgical management was still in its early stages during this period. The technique of aneurysm clipping, originally introduced by Dandy [9], was gradually refined, and intraoperative cerebral protection strategies began to appear in the literature. Botterell et al. [37] emphasized the importance of temporary arterial occlusion and intraoperative hypothermia in reducing ischemic injury during aneurysm clipping. These developments marked the transition of the “Intracranial Aneurysm Management” cluster from peripheral to basic relevance. The second area of research focused on aneurysm-related complications. The “Aneurysm Pathology” cluster, although still niche, expanded to include mass effect–related syndromes, as demonstrated by White et al. [38], who described compression of the pituitary stalk and cranial nerves. Motor themes also emerged regarding the etiology and prognosis of SAH. The seminal work of Hunt and Hess [6] showed that preoperative neurological status was the most important determinant of outcome, leading to the development of a clinical grading scale that remains influential today. Together, these advances reflect the transformation of aneurysm care from isolated surgical interventions to a more systematic approach that integrates diagnosis, pathology, and early risk stratification.

#### 4.2.2. Period 1971–1980

During 1971–1980, as illustrated in Figure 4b, the cluster “Aneurysm Surgical Techniques” shifts toward the peripheral and niche quadrants. The cluster encompassing “Cerebral Aneurysm Management” remains positioned between the basic and peripheral domains, although it gradually trends toward the niche and hot-topic zones. A new niche cluster, “Traumatic/Mycotic Aneurysms”, emerges during this time slice. In contrast, the cluster “Antifibrinolytic Aneurysm Therapy” represents the most prominent hot topic during this period.

This decade marked the transition into the microsurgical era, driven by the pioneering work of Yaşargil [10,11], who introduced the operating microscope and refined microsurgical instrumentation. These technological innovations redefined the surgical treatment of aneurysms, as reflected in the cluster “Aneurysm Surgical Techniques”, which, although categorized as a niche topic during this period, laid the groundwork for subsequent major advances in aneurysm management. Postoperative cerebral vasospasm emerged as a major focus of investigation. Weir et al. [39] demonstrated that vasospasm typically begins within three days of SAH and peaks between the sixth and eighth days. Building on these findings, Fisher et al. [40] established a correlation between the volume of subarachnoid blood and the severity of vasospasm, developing a CT-based prognostic grading system that achieved widespread clinical adoption. Collectively, these studies positioned vasospasm management at the forefront of motor research themes. Therapeutic strategies evolved in parallel. Kosnik and Hunt [41] reported that induced hypertension and hypervolemia improved cerebral perfusion in vasospastic territories, thereby reducing ischemic complications. Their findings contributed to elevating aneurysm management toward hot-topic status within the research landscape. Concurrently, a new niche cluster, “Traumatic/Mycotic Aneurysms”, emerged, highlighting rare but clinically challenging aneurysm subtypes. Ferguson [42] provided biomechanical evidence that aneurysm formation is influenced by hemodynamic stress, particularly at arterial bifurcations, an insight that proved foundational to the later rise of biomechanical research themes. Despite these advances, patient outcomes remained poor, with persistently high rates of morbidity and mortality following aneurysm rupture, as emphasized by Winn et al. [43]. Nevertheless, this decade firmly established modern surgical and pathophysiological concepts of IAs, bridging traditional approaches with emerging frameworks of risk-based and imaging-guided therapy.

#### 4.2.3. Period 1981–1990

Figure 4c depicts the time slice from 1981 to 1990. The topic “Aneurysm Imaging” remains a basic research area, with a marked increase in publication volume. “Cerebral Aneurysm Management” has transitioned into the basic-topics quadrant, reflecting its growing centrality. In contrast, “Aneurysm Surgical Techniques” has shifted toward the niche category, albeit as a relatively well-developed subfield. A newly identified and quantitatively substantial area, “Vasospasm Management”, appears during this period and rapidly evolves into a prominent hot topic. Simultaneously, a new neutral research domain labeled “Aneurysm Pathology” emerges, suggesting the early stages of thematic consolidation.

This period marked a major shift in IA research, characterized by the maturation of perioperative management strategies and the expansion of diagnostic and therapeutic imaging. The cluster “Aneurysm Imaging” remained a core and numerically dominant research domain, as digital subtraction angiography (DSA) became the standard imaging modality. Management of aneurysmal SAH was redefined by studies addressing natural history and treatment-related complications. Wiebers et al. [44] investigated the natural course of unruptured aneurysms and identified aneurysm size as a major risk factor for rupture. These insights consolidated “Cerebral Aneurysm Management” as an increasingly central basic research theme.

Several studies within the now well-developed cluster “Vasospasm Management” further advanced understanding of this critical complication. Grote et al. [45] examined the hyperacute phase of aneurysm rupture, Wijdicks et al. [46] analyzed perioperative natriuresis, and Voldby et al. [47] investigated cerebrovascular reactivity, together providing a deeper physiological framework. Kassell et al. [48] and Zubkov et al. [49] expanded the therapeutic potential of angiography, including the introduction of balloon angioplasty for vasospasm treatment. In addition, Petruk et al. [50] provided clinical evidence supporting the oral administration of nimodipine for vasospasm prevention, thereby establishing pharmacological vasospasm management as a prominent hot research area.

Meanwhile, the “Aneurysm Pathology” cluster re-emerged as a niche domain, focusing on aneurysm wall biology and surgical access techniques. These niche investigations incorporated explorations of novel microsurgical routes. Kawase et al.’s [51] transpetrosal approach and Dolenc’s [52] combined epidural–subdural approach to the carotico-ophthalmic region exemplified an increasing emphasis on surgical nuance and technical refinement. Collectively, these developments reflect a decade of consolidation, during which advances in imaging technology, perioperative physiological insight, and therapeutic refinement converged to transform aneurysm management from reactive intervention toward proactive, risk-stratified care.

#### 4.2.4. Period 1991–2000

The period from 1991 to 2000, as shown in Figure 4d, is characterized by the emergence of several distinct new clusters. Evidence-based medicine within the context of aneurysm management appears as a basic research theme. In parallel, a cluster centered on prognostic models in cerebral aneurysm management emerges at the intersection of basic and hot topics, indicating its increasing relevance. The domain of “Aneurysm Hemodynamics & Outcomes” is positioned at the boundary between peripheral and basic topics. Within the niche category, two well-developed clusters can be identified: one addressing complex surgical and endovascular management, and the other focusing specifically on endovascular aneurysm treatment.

This decade marked a pivotal evolution in IA research, characterized by the formal introduction of evidence-based medicine and an increasing emphasis on unruptured IAs. The cluster “Evidence-Based Aneurysm Management” emerged as a foundational research theme, reflecting growing awareness of the risks and benefits associated with prophylactic intervention. Rinkel et al. [1] estimated the prevalence of unruptured IAs to be approximately 2% among adults without SAH risk factors, with prevalence increasing with age. Juvela et al. [53] identified aneurysm size, patient age, and cigarette smoking as independent predictors of rupture. The influential ISUIA trial demonstrated that aneurysms smaller than 10 mm were associated with a low risk of rupture and that the morbidity and mortality related to treatment often did not justify intervention [31]. Collectively, these findings established unruptured IA management as a basic research theme transitioning toward hot-topic relevance.

Further refinement of risk stratification was provided by Ujiie et al. [54], who showed that rupture risk could be estimated on the basis of aneurysm shape and size, thereby introducing key morphological markers into clinical decision-making. On the pathophysiological front, the cluster “Aneurysm Pathology” continued to expand. Kataoka et al. [55] reported histological differences between ruptured and unruptured aneurysms, while Berendes et al. [56] identified salt-wasting syndrome and hyponatremia as complications in patients with SAH, broadening understanding of the systemic responses to aneurysm rupture.

A major innovation during this decade was the emergence of endovascular therapy, with the cluster “Endovascular Aneurysm Treatment” gaining prominence as a niche research theme. Guglielmi et al. [57,58] introduced electrothrombosis using electrically detachable coils, laying the foundation for modern coil embolization. These techniques represented a paradigm shift by offering a less invasive alternative to surgical clipping. In parallel, Lieber et al. [59] demonstrated that stent deployment could alter intra-aneurysmal hemodynamics, while ongoing computational modeling efforts began to quantify rupture risk based on flow dynamics. Although still peripheral at this stage, these studies signaled the impending centrality of biomechanical factors in aneurysm research. Overall, this decade bridged traditional morphological and clinical paradigms with emerging hemodynamic, radiologic, and endovascular concepts, preparing the field for the technological and conceptual advances of the twenty-first century.

#### 4.2.5. Period 2001–2010

Figure 4e depicts the time slice from 2001 to 2010. During this period, the cluster “Aneurysm Diagnosis & Treatment Strategies” constitutes the core set of basic research topics. The cluster “Aneurysm Risk Factors & Natural History” is positioned at the intersection of basic and hot topics, indicating increasing scientific attention. The cluster “Aneurysm Imaging & Diagnosis”, which had already demonstrated growth in earlier periods, matured into a highly developed and influential domain and is now classified among the hot topics of this time slice. The field of “Endovascular Treatment”, although not yet designated as a hot topic, moved substantially closer to this quadrant compared with the preceding period. A smaller niche cluster, “Compressive Aneurysm Treatment Management”, persisted during this interval. In contrast, the cluster “Hemodynamics & Biomechanics” shifted toward the peripheral zone, reflecting a relative decline in centrality compared with earlier periods.

The first decade of the twenty-first century witnessed a rapid expansion in both technical capabilities and conceptual frameworks within IA research. The cluster “Aneurysm Diagnosis & Treatment Strategies” consolidated as a central basic theme, reflecting a growing clinical emphasis on risk stratification and individualized therapeutic decision-making. Within the cluster “Aneurysm Risk Factors & Natural History”, epidemiological and clinical investigations advanced understanding of rupture predictors. Sonobe et al. [60], through the SUAVe study, provided population-level evidence indicating that aneurysms ≥4 mm in hypertensive patients younger than 50 years with multiple lesions warranted early intervention. These findings contributed to a more nuanced appreciation of rupture risk, thereby propelling this cluster toward hot-topic status.

Technological innovation was a defining feature of this period. The introduction of pipeline embolization devices (PEDs) represented a milestone in the evolution of endovascular therapy, now classified within the cluster “Endovascular Treatment”. Lylyk et al. [61] demonstrated the clinical utility of PEDs in treating large and wide-necked aneurysms, while Szikora et al. [62] reported successful parent artery reconstruction. The ISAT further accelerated this paradigm shift, concluding that endovascular coiling, in selected ruptured aneurysms amenable to both approaches, yielded superior short-term outcomes compared with surgical clipping. However, Piotin et al. [63] cautioned that stent-assisted coiling, although associated with reduced recurrence, might increase treatment-related mortality. In the context of unruptured IAs, Wermer et al. [64] contributed foundational insights by identifying demographic and aneurysmal characteristics associated with rupture.

The cluster “Hemodynamics & Biomechanics” gained prominence through studies by Shojima et al. [3] and Cebral et al. [65], which demonstrated the influence of wall shear stress (WSS) and complex intra-aneurysmal flow patterns on aneurysm development and rupture. Advanced imaging techniques also became integral clinical tools. Raabe et al. [66] introduced intraoperative indocyanine green videoangiography, enabling real-time assessment of flow dynamics and vessel patency, while Cebral et al. [67] developed image-based simulations for hemodynamic analysis, translating engineering principles into clinical practice. Finally, histological and genetic investigations broadened understanding of aneurysm biology. Frösen et al. [2] and Helgadottir et al. [68] explored the cellular and genetic underpinnings of aneurysm formation, bridging clinical phenotypes with molecular mechanisms. Overall, this decade was characterized by the convergence of biology, imaging, and engineering, resulting in a multidisciplinary integration that reshaped the diagnosis, evaluation, and treatment of aneurysms.

#### 4.2.6. Period 2011–2023

Figure 4f depicts the time slice from 2011 to 2023. During this period, the clusters related to aneurysm management and imaging consolidate within the basic research domain. Simultaneously, the previously niche cluster addressing complex aneurysm management shifts toward the peripheral quadrant. Endovascular therapy reverts to classification as a niche topic. The cluster “Natural Course of Unruptured Aneurysms” evolves into a more focused research area dedicated exclusively to unruptured aneurysms and attains a high level of development, now positioned among the hot topics. A newly detected cluster, “Biomechanics & Genetic Factors in the Development of Unruptured Aneurysms”, appears at the boundary between basic and hot topics. This location, together with its recent emergence, suggests growing research attention to this domain. Finally, the cluster representing treatment modalities remains situated at the interface between the hot and niche quadrants.

The most recent decade represents a period of consolidation and refinement in IA research, during which increasing clinical sophistication, multimodal imaging, and long-term outcome data have shaped contemporary management strategies. The cluster “Aneurysm Management & Imaging” emerged as a comprehensive basic research theme, reflecting the growing integration of diagnostic modalities with therapeutic planning. Improvements in the accessibility and quality of imaging have led to increased incidental detection of unruptured IAs. Vlak et al. [33] estimated a general population prevalence of 3.2%. In response to the growing need for individualized rupture-risk assessment, Greving et al. [4] introduced the PHASES score, which incorporates population, hypertension, age, aneurysm size, previous SAH, and aneurysm location. Building on this work, Backes et al. [69] proposed the Earlier subarachnoid hemorrhage, Location of aneurysm, Age > 60 years, Population, Size of aneurysm, Shape of aneurysm (ELAPSS) score to predict aneurysm growth, emphasizing morphological characteristics, particularly aneurysm shape. These tools underscore the rising influence of the cluster “Natural Course of Unruptured Aneurysms”, which attained hot-topic status during this period. The Unruptured Cerebral Aneurysm Study (UCAS) Japan study further demonstrated that aneurysm size, location, and morphology significantly affect rupture risk [70]. Xiang et al. [71] and Meng et al. [72] extended these findings by identifying associations between hemodynamic factors, particularly low WSS, and inflammatory cell-mediated remodeling of aneurysm walls, thereby differentiating aneurysm subtypes according to distinct rupture mechanisms.

Parallel advances occurred within the newly emerged cluster “Biomechanics & Genetic Factors in the Development of Unruptured Aneurysms”. Meng et al. [72] distinguished between atherosclerotic-type aneurysms, associated with low WSS and inflammatory pathways, and secondary bleb-type aneurysms, associated with high WSS. Chalouhi et al. [73] confirmed that inflammation contributes to aneurysm growth and rupture and proposed inflammatory pathways as potential therapeutic targets.

Moreover, treatment modalities and outcomes were extensively evaluated during this decade. The debate evolved from a dichotomous “clip versus coil” paradigm to a more nuanced “clip and coil” approach. Steiner et al. [74], in the European Stroke Organisation (ESO) guidelines, emphasized individualized treatment decisions based on aneurysm- and patient-specific characteristics. Long-term follow-up data from the ISAT and the Barrow Ruptured Aneurysm Trial (BRAT) provided further insights. Molyneux et al. [75] reported higher long-term mortality in the clipping group and higher rebleeding rates in the coiling group over 18 years of follow-up. Spetzler et al. [76], in the BRAT study, demonstrated lower retreatment rates with surgical management, alongside substantial cross-over between treatment arms.

The cluster “Endovascular Therapy & Devices” remained highly active, marked by continued innovation. McDougall et al. [77] presented one-year BRAT data favoring coiling in terms of fewer poor clinical outcomes. Hong et al. [78] reported that stent-assisted coiling was superior to conventional coiling for small berry aneurysms. Nelson et al. [79] and Kallmes et al. [80] confirmed the safety and feasibility of flow diverters, such as the PED, in treating wide-necked and previously failed aneurysms, particularly in the internal carotid artery. However, Cebral et al. [81] cautioned that PED-induced hemodynamic alterations may precipitate rupture in specific aneurysm subtypes, and Brinjikji et al. [82] highlighted that, despite their benefits, flow-diverting devices carry non-negligible procedural risks. Chalouhi et al. [83] further demonstrated that stent-assisted coiling reduces recanalization rates and procedural complications. In long-term BRAT follow-up, Spetzler et al. [76] showed superior obliteration rates with microsurgical clipping, particularly for anterior circulation aneurysms, whereas endovascular embolization yielded more favorable outcomes for posterior circulation lesions.

Taken together, the thematic landscape of this period reflects the consolidation of an increasingly integrated and multidisciplinary research agenda. From personalized risk stratification to image-guided therapy and biologically informed prognostication, the thematic profile points to growing interest in precision-medicine approaches. Whether this research attention translates into clinical effectiveness and routine practice cannot be determined from the present analysis.

#### 4.2.7. Thematic Evolution

**Figure 5:**
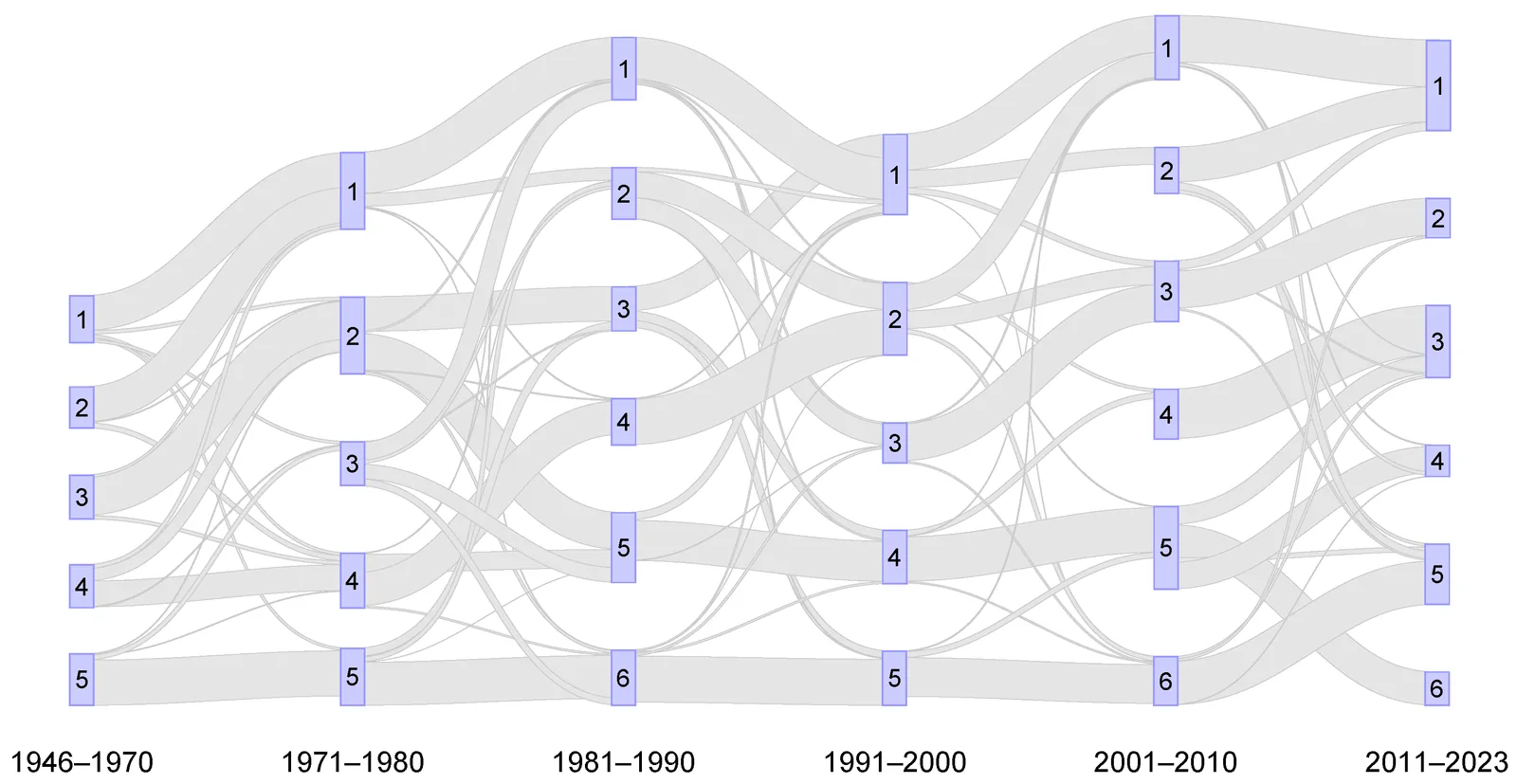
Alluvial diagram depicting the thematic evolution of intracranial aneurysm research from 1946 to 2023. The diagram traces the longitudinal continuity, divergence, and transformation of thematic clusters identified through co-word analysis across six consecutive analytical periods. Each node represents a thematic cluster, with full cluster labels provided in Table 2. Node height is proportional to the number of MeSH terms contained within the cluster. Alluvia connecting nodes across adjacent periods represent thematic transitions, and their width is proportional to the thematic stability index between periods. The three flow patterns discussed in the text are distinguished by topology rather than by color: continuity appears as a single dominant alluvium linking a cluster to one successor of similar composition; divergence as outgoing alluvia splitting into two or more successor clusters; and transformation as a cluster that persists but is relabeled owing to a substantial change in term composition.

Figure 5 reveals several consistent longitudinal flow patterns in the thematic evolution of IA research, where flow continuity reflects the degree of shared MeSH term overlap between adjacent periods. The flow patterns in the alluvial diagram (continuity, divergence, and transformation) are distinguished by the topology of the alluvia (single, splitting, or relabeled flows, respectively), rather than by color; given the number of clusters and periods involved, the diagram is necessarily dense, and the text below highlights the pathways of greatest thematic and clinical relevance rather than an exhaustive account of every alluvium. It presents a clear evolutionary path in IA research, characterized by progressive consolidation toward endovascular approaches and specialized management strategies.

A prominent longitudinal pathway tracks the emergence of endovascular approaches. This pathway extends from “IA Management” (1946–1970, Cluster 1) and “Angiographic Findings in Vascular Anomalies” (1946–1970, Cluster 2), passes through “Vasospasm Management” (1981–1990, Cluster 2), and leads to the newly differentiated “Endovascular Aneurysm Treatment” (1991–2000, Cluster 3), which retains longitudinal thematic continuity across the final two periods from 2001 to 2023 (Clusters 2 and 3, respectively). Endovascular approaches also integrate into “Surgical & Endovascular Complex Aneurysm Management” (1991–2000, Cluster 5). This cluster emerges from the evolutionary flow of “Aneurysm Surgical Techniques”, a domain traditionally dominated by microsurgical strategies (Cluster 5 in 1946–1980 and Cluster 6 in 1981–1990). In addition, “Endovascular Therapy & Devices” (2011–2023, Cluster 6) becomes integrated into the hemodynamic and biomechanical research flow originating from “Hemodynamics & Biomechanics” (2001–2010, Cluster 5), reflecting the clinical implementation of new endovascular devices.

A second major evolutionary pattern is the emergence of mechanistic and risk-oriented research domains in the later periods. “Hemodynamics & Biomechanics” (2001–2010, Cluster 5) develops as a distinct downstream continuation of prior treatment-oriented clusters and remains connected to “Biomechanics & Genetics in Unruptured IA Development” (2011–2023, Cluster 4), reflecting increased academic attention to aneurysm behavior, rupture susceptibility, and individualized risk assessment. In parallel, a dedicated longitudinal pathway culminates in “Unruptured Aneurysm Natural Course, Diagnosis & Management” (2011–2023, Cluster 1), indicating the consolidation of unruptured IA management as an autonomous thematic subdomain.

Taken together, the alluvial structure indicates a gradual transition from heterogeneous early emphases on surgical techniques, angiographic characterization, and pathology toward specialized, interconnected themes centered on evidence-based medicine. The current research focus has firmly converged on endovascular intervention, biomechanical modeling, and risk-stratified management of unruptured aneurysms for personalized IA treatment.

## 5. Discussion

The present knowledge mapping synthesizes eight decades of conceptual and technological developments in IA research. Analysis of more than 22,000 publications demonstrates a progressive shift in research focus from angiographic characterization and microsurgical clipping toward a multidisciplinary landscape emphasizing endovascular intervention, computational hemodynamics, and individualized rupture risk assessment. These findings both corroborate and extend previous bibliometric investigations, highlighting the evolving thematic complexity of the field.

For interpretive clarity, we can trace four broader historical eras across the six co-word analysis periods: open-surgery, transitional, endovascular, and multidisciplinary. These eras reflect predominant clinical and technological themes and represent an interpretative synthesis of the six predefined time periods rather than four independently identified bibliometric clusters; the co-word analysis itself was performed separately for each period. The temporal patterns capture the progression from microsurgical refinement and advances in perioperative care to evidence-based decision frameworks and the widespread adoption of endovascular techniques. They are consistent with the publication trends documented by Kiraz et al. [12], who identified exponential growth beginning in the 1980s and sustained productivity concentrated in the United States, Japan, and China. The successive rise of themes related to endovascular coiling and subsequently to flow-diverting devices is evident in our thematic evolution diagrams, consistent with previous bibliometric analyses [16,84]. Landmark trials, notably ISAT and ISUIA, mark the early 2000s as a pivotal inflection point preceding the contemporary evidence-based era, in which multidisciplinary and individualized treatment algorithms integrate endovascular and microsurgical strategies to optimize care for patients with IAs.

Increased attention to unruptured IA management reflects the trends noted in prior studies. Lu et al. [15] attributed this shift to implementation of prognostic tools such as the PHASES score; our later time periods similarly demonstrate growing emphasis on personalized rupture risk estimation and natural history modeling. Concurrently, hemodynamic research has expanded considerably in recent years. We observed hemodynamic modeling emerging as a dominant theme, consistent with the characterization of computational hemodynamics research by Chen et al. [22]. Their documentation of computational fluid dynamics, 4D-flow MRI, and fluid–structure interaction as central to understanding aneurysm biology corresponds with the increasing prominence of these topics within the thematic clusters identified in our analysis. The evolution from purely morphological parameters toward integrated hemodynamic–morphologic signatures in rupture prediction, as described by Zheng et al. [85], Zhang et al. [16], and Qin et al. [17], is similarly evident in our temporal analysis.

Our results complement previous observations regarding the limited representation of fundamental science. Although biological and pathophysiological themes appear in early periods, mechanistic investigation remains proportionally constrained in recent decades. Analyses of animal models by Chen et al. [19] and longitudinal assessments by Zhang et al. [23] both indicate that mechanistic research has comprised less than one-fifth of publications since 2000, despite its essential role in therapeutic development. This disparity highlights a persistent translational deficit requiring strategic attention.

Consistent with bibliometric analyses of aneurysmal SAH, our findings indicate that clinical management is a dominant theme but relies heavily on observational data. Borsatto et al. [86] emphasized the paucity of randomized controlled trials in SAH research. Our thematic mapping confirms that despite extensive clinical literature, substantial uncertainty persists regarding optimal peri-interventional protocols, vasospasm management, and long-term monitoring of treated versus untreated lesions. This evidence gap reflects and perpetuates the continued prominence of prognostic modeling and outcome prediction research.

Since the early 2010s, advances in AI have reshaped scientific research, with computational methods moving from peripheral tools to integral components of data-driven discoveries [87]. Machine learning-related terms appear prominently in our most recent period, reflecting the rapid expansion documented by Zhang et al. [23], who noted a tenfold increase in AI-focused aneurysm publications over the past decade. Our co-word analysis captures this rising bibliometric prominence of AI-related themes but does not, by itself, assess methodological quality or clinical performance; the accuracy figures for segmentation and rupture prediction reported elsewhere in the literature point to considerable potential, but rigorous external validation and prospective clinical implementation remain necessary before this prominence can be interpreted as established clinical value.

Several methodological considerations merit acknowledgment. Our analysis exclusively encompasses MEDLINE/PubMed-indexed English-language publications, potentially introducing geographic and linguistic selection bias and underrepresenting contributions indexed in alternative databases, including Scopus or Web of Science. Reliance on MeSH headings as analytic units introduces inherent variability, as indexing practices and keyword assignments differ across authors, journals, and historical periods. While our methodology incorporates frequency thresholds to minimize the impact of infrequent terms, biases related to indexing conventions remain unavoidable. Co-word analysis characterizes thematic relationships but does not evaluate the methodological quality, clinical significance, or translational value of individual studies. The six analytical periods also differ substantially in duration and publication volume; in particular, the final period (2011–2023) spans 13 years and contains far more documents than any earlier slice (Table 2). This uneven partitioning represents a methodological trade-off, as extending the final window to 2023 avoided creating a short residual period with too few publications for reliable co-word mapping, a pragmatic approach in longitudinal bibliometric studies. While this imbalance mirrors the genuine growth trajectory of the field and does not invalidate the analysis, it likely affects thematic complexity and limits direct comparability of centrality and density values across periods, since larger, more recent corpora can support a greater number of more finely differentiated clusters than earlier, sparser periods. Although the temporal partitioning approach is necessary for longitudinal assessment, it may obscure the gradual thematic evolution that occurs within decadal intervals. Finally, an emphasis on publication patterns rather than citation networks constrains insights regarding knowledge dissemination and intellectual influence. Notwithstanding these limitations, our dataset, which exceeds 22,000 publications, provides substantial quantitative evidence of thematic development and yields valuable perspectives for strategic research planning in IA investigation.

Future work should address these constraints using complementary methodological strategies. Integration of citation network analysis and author collaboration mapping would clarify knowledge transfer pathways and identify influential research communities beyond thematic relationships. The application of text mining and natural language processing to abstracts and full-text content could mitigate inconsistencies in author-assigned keywords and MeSH indexing, revealing latent conceptual structures not detected through the presented co-word approach. Qualitative methodologies, including systematic literature synthesis and expert panel consultations, would contextualize our quantitative observations within broader clinical, technological, and policy contexts. Additionally, comparative analyses across subdisciplines (i.e., distinguishing between ruptured and unruptured IA or surgical and endovascular management) would clarify domain-specific evolutionary patterns. Finally, prospective mapping investigations should monitor emerging themes identified in our recent period, particularly AI applications, patient-centered outcomes, and precision medicine strategies, to evaluate their maturation and incorporation into standard clinical practice while informing the strategic prioritization of research investment.

## Figures and Tables

**Table 1:** The 10 most-cited articles in IA research.

Title	TC	TC/Y	NTC
Unruptured intracranial aneurysms: Natural history, clinical outcome, and risks of surgical and endovascular treatment [27]	2635	119.8	54.3
International Subarachnoid Aneurysm Trial (ISAT) of neurosurgical clipping versus endovascular coiling in 2143 patients with ruptured intracranial aneurysms: A randomised trial [28]	2605	113.3	52.2
Surgical risk as related to time of intervention in the repair of intracranial aneurysms [6]	2584	45.3	35.1
Validity and reliability of a quantitative computed tomography score in predicting outcome of hyperacute stroke before thrombolytic therapy [29]	1830	73.2	37.1
The International Cooperative Study on the Timing of Aneurysm Surgery. Part 1: Overall management results [30]	1502	42.9	26.5
Unruptured intracranial aneurysms–risk of rupture and risks of surgical intervention [31]	1286	47.6	28.1
Subarachnoid haemorrhage [32]	1242	69.0	32.9
Prevalence of unruptured intracranial aneurysms, with emphasis on sex, age, comorbidity, country, and time period: A systematic review and meta-analysis [33]	1199	85.6	36.2
Long-term angiographic recurrences after selective endovascular treatment of aneurysms with detachable coils [34]	1135	51.6	23.4
Prevalence and risk of rupture of intracranial aneurysms: A systematic review [1]	1073	39.7	23.5

Legend: TC, total citations; TC/Y, total citations per year; NTC, normalized total citations (calculated by dividing an article’s total citations by the mean citations of all articles published in the same year).

**Table 2:** Thematic clusters and structural characteristics identified by co-word analysis.

Period	Clust	*n*	Docs	Cent	Dens	Cluster Label
1946–1970	1	39	174	2.84	38.08	Intracranial Aneurysm Management
	2	51	48	6.75	48.41	Angiographic Findings in Vascular Anomalies
	3	36	62	2.37	71.01	Aneurysms & Comorbidities
	4	21	100	0.91	60.16	Aneurysm Pathology
	5	51	197	4.90	52.67	Aneurysm Surgical Techniques
1971–1980	1	44	229	2.17	18.75	Cerebral Aneurysm Management
	2	40	254	2.51	15.79	Aneurysm Imaging Techniques
	3	53	176	2.23	21.27	Antifibrinolytic Aneurysm Therapy
	4	44	259	1.83	24.95	Traumatic/Mycotic Aneurysms
	5	19	81	0.83	20.74	Aneurysm Surgical Techniques
1981–1990	1	47	47	1.47	11.09	Cerebral Aneurysm Management
	2	54	359	1.61	13.77	Vasospasm Management
	3	48	208	1.92	10.96	Aneurysm Imaging
	4	20	400	0.70	13.53	Complex Aneurysm Management & Endovascular Aneurysm Treatment
	5	23	128	0.83	11.33	Aneurysm Pathology
	6	8	450	0.35	12.63	Aneurysm Surgery Techniques
1991–2000	1	46	589	1.22	5.83	Cerebral Aneurysm Prognostic Models & Management
	2	41	483	1.38	4.95	Evidence-Based Approach for Aneurysm Management/Treatment
	3	46	593	0.86	6.00	Endovascular Aneurysm Treatment
	4	35	285	0.94	5.15	Aneurysm Hemodynamics & Outcomes
	5	32	567	0.68	8.92	Surgical & Endovascular Complex Aneurysm Management
2001–2010	1	52	277	1.31	4.81	Aneurysm Imaging & Diagnosis
	2	41	821	0.82	3.50	Aneurysm Diagnosis & Treatment Strategies
	3	44	974	0.71	3.99	Endovascular Treatment/Strategies
	4	42	144	0.78	3.80	Aneurysm Risk Factors & Natural History
	5	12	855	0.17	3.19	Hemodynamics & Biomechanics
	6	9	818	0.19	4.07	Comprehensive Aneurysm Treatment Management
2011–2023	1	59	1946	0.36	2.18	Unruptured Aneurysm Natural Course, Diagnosis & Management
	2	29	1521	0.27	1.59	Imaging & Management
	3	30	182	0.25	1.81	Treatment Modalities & Outcomes
	4	48	1415	0.36	2.18	Biomechanics & Genetics in UIA Development
	5	27	2478	0.16	1.79	Treatment Modalities & Outcomes in Complex Aneurysms
	6	7	2070	0.08	2.18	Endovascular Therapy & Devices

Legend: Clust, cluster identifier; *n*, no. of unique MeSH terms in the cluster; Docs, no. of documents assigned to the cluster; Cent, centrality; Dens, density.

## Data Availability

The programming code used to retrieve the data and reproduce the results is publicly available in the GitHub repository at https://github.com/akastrin/neurobib (accessed on 11 September 2026).

## Abbreviations

The following abbreviations are used in this manuscript:

4D-MRI	four-dimensional flow magnetic resonance imaging
AI	artificial intelligence
ASPECTS	Alberta Stroke Programme Early CT Score
BRAT	Barrow Ruptured Aneurysm Trial
DSA	digital subtraction angiography
ELAPSS	Earlier subarachnoid hemorrhage, Location of aneurysm, Age > 60 years, Population,
	Size of aneurysm, Shape of aneurysm
ESO	European Stroke Organisation
IA	intracranial aneurysm
ISAT	International Subarachnoid Aneurysm Trial
ISUIA	International Study of Unruptured Intracranial Aneurysms
MeSH	Medical Subject Headings
NLM	US National Library of Medicine
PED	pipeline embolization device
PHASES	Population, Hypertension, Age, Size of aneurysm, Earlier SAH, Site of aneurysm
SAH	subarachnoid hemorrhage
UCAS	Unruptured Cerebral Aneurysm Study
WSS	wall shear stress
